# Reductive evolution and the loss of PDC/PAS domains from the genus *Staphylococcus*

**DOI:** 10.1186/1471-2164-14-524

**Published:** 2013-07-31

**Authors:** Neethu Shah, Rosmarie Gaupp, Hideaki Moriyama, Kent M Eskridge, Etsuko N Moriyama, Greg A Somerville

**Affiliations:** 1 Department of Computer Science and Engineering, University of Nebraska, Lincoln, NE 68588-0115, USA; 2 School of Veterinary Medicine and Biomedical Sciences, University of Nebraska, Lincoln, NE 68583-0905, USA; 3 School of Biological Sciences, University of Nebraska, Lincoln, NE 68588-0118, USA; 4 Department of Statistics, University of Nebraska, Lincoln, NE 68583-0963, USA; 5 School of Biological Sciences and Center for Plant Science Innovation, University of Nebraska, Lincoln, NE 68588-0118, USA

**Keywords:** Staphylococcus, PAS domain, Molecular evolution, Phylogeny

## Abstract

**Background:**

The Per-Arnt-Sim (PAS) domain represents a ubiquitous structural fold that is involved in bacterial sensing and adaptation systems, including several virulence related functions. Although PAS domains and the subclass of PhoQ-DcuS-CitA (PDC) domains have a common structure, there is limited amino acid sequence similarity. To gain greater insight into the evolution of PDC/PAS domains present in the bacterial kingdom and staphylococci in specific, the PDC/PAS domains from the genomic sequences of 48 bacteria, representing 5 phyla, were identified using the sensitive search method based on HMM-to-HMM comparisons (HHblits).

**Results:**

A total of 1,007 PAS domains and 686 PDC domains distributed over 1,174 proteins were identified. For 28 Gram-positive bacteria, the distribution, organization, and molecular evolution of PDC/PAS domains were analyzed in greater detail, with a special emphasis on the genus *Staphylococcus*. Compared to other bacteria the staphylococci have relatively fewer proteins (6–9) containing PDC/PAS domains. As a general rule, the staphylococcal genomes examined in this study contain a core group of seven PDC/PAS domain-containing proteins consisting of WalK, SrrB, PhoR, ArlS, HssS, NreB, and GdpP. The exceptions to this rule are: 1) *S. saprophyticus* lacks the core NreB protein; 2) *S. carnosus* has two additional PAS domain containing proteins; 3) *S. epidermidis*, *S. aureus*, and *S. pseudintermedius* have an additional protein with two PDC domains that is predicted to code for a sensor histidine kinase; 4) *S. lugdunensis* has an additional PDC containing protein predicted to be a sensor histidine kinase.

**Conclusions:**

This comprehensive analysis demonstrates that variation in PDC/PAS domains among bacteria has limited correlations to the genome size or pathogenicity; however, our analysis established that bacteria having a motile phase in their life cycle have significantly more PDC/PAS-containing proteins. In addition, our analysis revealed a tremendous amount of variation in the number of PDC/PAS-containing proteins within genera. This variation extended to the *Staphylococcus* genus, which had between 6 and 9 PDC/PAS proteins and some of these appear to be previously undescribed signaling proteins. This latter point is important because most staphylococcal proteins that contain PDC/PAS domains regulate virulence factor synthesis or antibiotic resistance.

## Background

Basic cellular functions (*e.g.*, DNA replication and transcription) are regulated in response to environmental and nutritional signals. For this reason, sensing and transduction of these extracellular and intracellular signals is of critical importance for survival. Many of the signaling and regulatory proteins involved in responding to environmental cues have a modular composition of specialized domains [1,2]. One of these specialized domains is the Per-Arnt-Sim (PAS) domain, which is distributed throughout all genera of life. PAS domains are identified by particular patterns of secondary structures and they have important roles as sensory modules for environmental and nutritional signals, such as voltage, gases, light, metabolites or redox potential [3-5]. In addition to regulating physiological processes, several PAS domain containing proteins are involved in regulating virulence determinants in pathogenic bacteria [6-10]. The wide-distribution of PAS domains, the diversity of signals they transduce, and the array of regulatory pathways in which they are involved are reflected in their extremely divergent amino acid sequences [11-13]. Our interest in PAS domain-containing proteins is due to their involvement in sensing and responding to changes in concentrations of dicarboxylic acids [14].

Bacterial survival depends on their ability to regulate transcription of genes in response to changing environmental and nutritional conditions. In pathogenic bacteria, the response to changes in nutritional or environmental conditions often includes activating transcription of virulence genes [15]. The genus *Staphylococcus* includes both pathogenic and non-pathogenic species that live in diverse environmental conditions such as the anterior nares (*i.e*., *S. aureus*), the skin (*i.e.*, *S. epidermidis*), and in dry sausages (*i.e.*, *S. carnosus*) [16]. The medical and economic importance of staphylococci has generated considerable interest in how these bacteria “sense” and respond to changes in their environment. In part, the medical interest stems from a desire to control staphylococcal infections, while the commercial interests involve understanding the role of primary and secondary metabolites in flavoring fermented meats. This interest in environmental sensing and the importance of PAS domains in regulating responses to environmental and nutritional signals led us to examine eight genomes of seven staphylococcal species for the presence of PAS domains. Specifically, we wanted to identify potential dicarboxylic acid sensing proteins that could link changes in tricarboxylic acid cycle activity to transcriptional alterations that affect virulence factor synthesis [15]. To gain insight into the potential functions and evolutionary relationships of these domains, we compared the staphylococcal PAS domains to those of a diverse group (47 different species) of bacteria representing five phyla.

**Figure 1:**
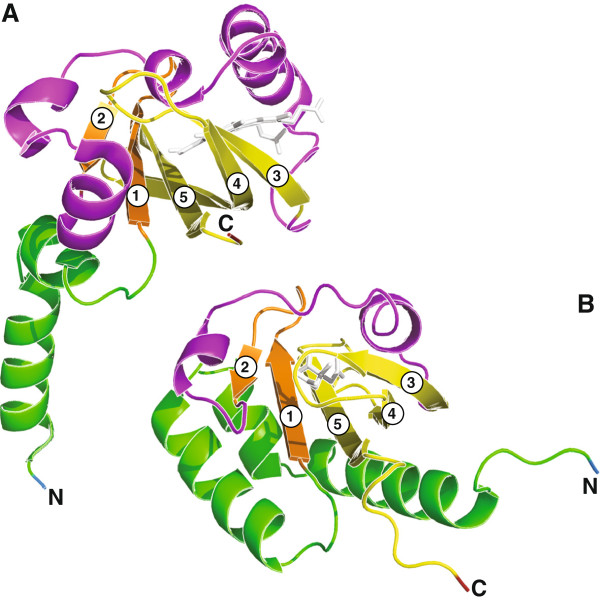
**Three-dimensional structures of PAS (A) and PDC (B) domains.** The structures are based on **(A)** the *Rhizobium meliloti* oxygen sensor FixL protein with its ligand heme (UniProt P10955: positions 122–251) (PDB: 1D06) [21], and **(B)** the ligand-binding domain of the *Klebsiella pneumoniae* sensor kinase CitA protein with its ligand citrate (UniProt P52687: positions 5–135) (PDB: 1P0Z) [22]. For both structures, the core β strands are labeled from 1 to 5. Schematic models were generated by PyMol (Schrödinger, Portland, OR). Each region is colored as follows: the amino end with blue, the leading α-helix region with green, the first two β-strands with orange, the inter-domain α-helix region with magenta, the last three β-strands with yellow, and the carboxyl end with red. Ligands are shown in white stick models.

PAS domains are typically between 100–120 amino acids in length; however, some are larger [4,11,17]. The core of a PAS domain is a five stranded β-sheet, and interspersed within this core are α-helices that provide ligand/signal specificity [4,18]. The position of α-helices can vary depending on the cellular location; specifically, cytoplasmic PAS domains have an α-2β-4α-3β topology (Figure 1A) and extracytoplasmic PAS domains have a 3α-2β-1/2α-3β-α topology (Figure 1B) [13,18,19]. These extracytoplasmic PAS domains are typified by the PhoQ, DcuS, and CitA proteins, and are referred to as PDC domains [20].

PAS domains share less than 20% amino acid sequence identity [4,11-13]; hence, identifying PDC/PAS domain containing proteins across diverse genomes has been difficult. Taylor and Zhulin [4] used position-specific iterative BLAST (PSI-BLAST) [23] to identify 68 PAS-containing proteins from eleven bacterial genomes. Of the eleven genomes, only six were found to have PAS-containing proteins and several of these proteins included multiple PAS domains. An analysis of sixteen bacterial species using PSI-BLAST similarity search revealed a wide distribution of small-molecule binding domains, including PAS domains, in signal transducing proteins, transporters, and enzymes [1]. In a search of PAS domain containing proteins in two genomes of *Cyanobacteria* (*i.e.*, *Nostoc punctiforme* and *Anabena* sp.), Narikawa *et al.*[24] used profile hidden Markov models (HMMs) to identify a total of 323 PAS domains. More recently, using the fold and function assignment system (FFAS) algorithm [25], Chang *et al.*[19] found that eleven out of thirteen sensor kinases in the *Bacillus subtilis* genome possessed PDC domains.

In order to expand our understanding of PDC and PAS domain distribution in bacteria and their evolutionary history, we chose to use more sensitive search methods, on a more diverse array of bacterial genomes. Specifically, we used the recently developed sensitive search method based on HMM-to-HMM comparisons (HHblits) [26]. Using this approach, we identified a total of 1,174 PDC/PAS domain containing proteins (for brevity, we call them PDC/PAS proteins henceforth) in 48 diverse bacterial genomes, including both Gram-negative and Gram-positive bacteria. The number of PDC/PAS proteins varied across these genomes (ranging from 3 to 98 proteins) and more PAS domains (1,007) were identified than PDC domains (686). Interestingly, the number of PDC/PAS proteins also varied among the eight staphylococcal genomes; as an example, *Staphylococcus saprophyticus* had six PDC/PAS proteins, while *Staphylococcus carnosus* had nine. Lastly, these data provided an opportunity to examine the evolution and function of staphylococcal PDC/PAS proteins.

## Results and discussion

### PDC/PAS domains in evolutionarily diverse bacteria

**Figure 2:**
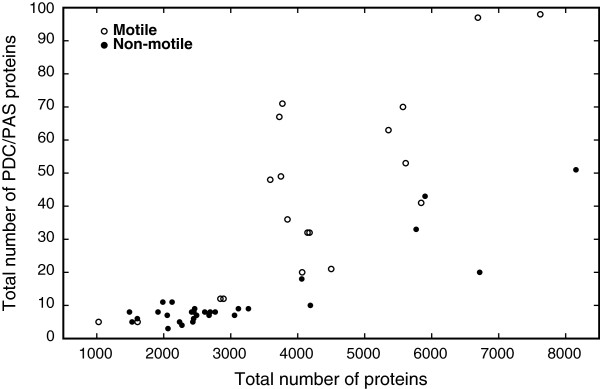
**Correlation between the total protein numbers and the numbers of PDC/PAS proteins across 48 bacterial genomes.** The correlation is significant either based on all 48 genomes or based on only 43 genomes (excluding 5 over-representing *Staphylococcus* genomes): for 48 genomes, Pearson’s correlation coefficient *r* = 0.77 (*p* < 0.0001) and Spearman’s rank correlation *ρ* = 0.83 (*p* < 0.0001); for 43 genomes, Pearson’s correlation coefficient *r* = 0.76 (*p* < 0.0001) and Spearman’s rank correlation *ρ* = 0.82 (*p* < 0.0001). Bacterial species were classified as motile or non-motile (see Table 1), and they were plotted with open and closed circles, respectively.

The importance of PAS domains in bacterial signal transduction systems and the abundance of staphylococcal genomic DNA sequences provided an excellent opportunity to analyze staphylococcal genomes for the presence of PDC and PAS domains. In order to understand the molecular evolution of staphylococcal PDC and PAS domains within the bacterial kingdom, we identified these domains from a diverse set of 48 bacterial genomes, including seven species of *Staphylococcus* (Table 1). Due to the availability of multiple *S. aureus* genomes and their medical importance, two strains (COL and USA300_TCH1516) of *S. aureus* were included in the analyses. The domains were classified based on the predicted secondary structure as PAS (α-2β-4α-3β) or PDC (3α-2β-1/2α-3β-α) folds (Table 1 and found in Additional file 1: Table S1). A total of 1,693 PAS/PDC domains in 1,174 proteins were identified, of which 1,007 were PAS folds and 686 domains were PDC folds. Consistent with the ubiquitous nature of PAS domains, all bacteria examined had PAS domains. Similarly, PDC domains were found in all genomes examined except for *Lactococcus lactis*. As previously observed [24], the filamentous cyanobacterium *Nostoc punctiforme* had the greatest number of total PDC/PAS domains (169 domains distributed over 97 proteins), as well as the greatest number of PAS domains (131 domains) while *Vibrio cholerae* had the most PDC domains (59 domains) (Table 1). In general, smaller bacterial genomes had fewer PDC/PAS proteins, hence fewer PDC/PAS domains, while larger genomes had more PDC/PAS proteins and more PDC/PAS domains. To determine if a correlation existed between the total number of proteins and PAS domains, we calculated a correlation coefficient for these two variables. As expected, a significant positive correlation was found between the total number of proteins and the number of PDC/PAS proteins (Figure 2; Pearson's correlation coefficient *r* = 0.76, *p* < 0.0001; Spearman’s rank correlation *ρ* = 0.82, *p* < 0.0001).

**Table 1:** Summary of the 48 bacterial genomes used in this study and the PAS/PDC domains identified

**Species**^**a**^	**Subspecies/strain**	**Accession number**	**Phylum**	**Pathogenicity**^**b**^	**Motility**^**c**^	**Total proteins**^**d**^	**PDC/PAS proteins**^**e**^	**PAS domains**^**f**^	**PDC domains**^**g**^
**[Gram positive]**									
*Bifidobacterium animalis*	*lactis* AD011	NC_011835.1	Actinobacteria	0	0	1527	5	4	2
*Corynebacterium diphtheriae* (Ci)	NCTC 13129	NC_002935.2	Actinobacteria	1	0	2272	4	2	2
*Corynebacterium glutamicum* (Cg)	ATCC 13032	NC_006958.1	Actinobacteria	0	0	3057	7	5	4
*Micrococcus luteus* (Ml)	NCTC 2665	NC_012803.1	Actinobacteria	0	0	2236	5	4	3
*Mycobacterium leprae*	TN	NC_002677.1	Actinobacteria	1	0	1605	6	7	1
*Mycobacterium smegmatis* (Ms)	MC2 155	NC_008596.1	Actinobacteria	0	0	6717	20	17	10
*Mycobacterium tuberculosis* (Mt)	CDC1551	NC_002755.2	Actinobacteria	1	0	4189	10	6	7
*Streptomyces coelicolor* (Stc)	A3(2)	NC_003888.3	Actinobacteria	0	0	8154	51	48	18
*Bacillus anthracis* (Ba)	CDC 684	NC_012581.1	Firmicutes	1	0	5902	43	30	30
*Bacillus cereus* (Bc)	ATCC 10987	NC_003909.8	Firmicutes	1	1	5843	41	30	25
*Bacillus megaterium* (Bm)	QM B1551	NC_010010.2	Firmicutes	0	1	5612	53	57	21
*Bacillus subtilis* (Bs)	*subtilis* 168	NC_000964.3	Firmicutes	0	1	4176	32	21	30
*Clostridium acetobutylicum* (Ca)	ATCC 824	NC_003030.1	Firmicutes	0	1	3847	36	16	34
*Clostridium botulinum*	A str. ATCC 3502	NC_009496.1	Firmicutes	1	1	3590	48	31	40
*Clostridium difficile* (Cd)	630	NC_009089.1	Firmicutes	1	1	3749	49	49	26
*Enterococcus faecalis* (Efs)	V583	NC_004668.1	Firmicutes	1	0	3264	9	7	7
*Enterococcus faecium* (Efm)	DO	NZ_AAAK00000000	Firmicutes	1	0	3114	9	3	10
*Lactobacillus casei* (Lc)	ATCC 334	NC_008526.1	Firmicutes	0	0	2768	8	6	4
*Lactococcus lactis* (Ll)	*cremoris* MG1363	NC_009004.1	Firmicutes	0	0	2434	5	5	0
*Listeria monocytogenes* (Lm)	EGD-e	NP_463535.1	Firmicutes	1	1	2846	12	9	7
*Macrococcus caseolyticus* (Mc)	JCSC5402	NC_011999.1	Firmicutes	0	0	2052	7	6	4
*Staphylococcus aureus* (SaU)	*aureus* USA300_TCH1516	NC_010079.1	Firmicutes	1	0	2693	8	5	7
*Staphylococcus aureus* (SaC)	*aureus* COL	NC_002951.2	Firmicutes	1	0	2612	8	5	7
*Staphylococcus carnosus* (Sc)	*carnosus* TM300	NC_012121.1	Firmicutes	0	0	2461	9	8	5
*Staphylococcus epidermidis* (Se)	ATCC 12228	NC_004461.1	Firmicutes	1	0	2416	8	5	7
*Staphylococcus haemolyticus* (Sh)	JCSC1435	NC_007168.1	Firmicutes	1	0	2676	7	5	4^h^
*Staphylococcus lugdunensis* (Sl)	HKU09-01	CP_001837.1	Firmicutes	1	0	2490	7	5	5
*Staphylococcus pseudintermedius* (Sp)	HKU10-03	NC_014925.1	Firmicutes	1	0	2450	8	5	7
*Staphylococcus saprophyticus* (Ss)	*saprophyticus* ATCC15305	NC_007350.1	Firmicutes	1	0	2446	6	4	5
*Streptococcus agalactiae*	2603 V/R	NC_004116.1	Firmicutes	1	0	2124	11	6	7
*Streptococcus pneumoniae* (Spn)	D39	NC_008533.1	Firmicutes	1	0	1914	8	5	4
*Streptococcus pyogenes* (Spy)	MGAS10270	NC_008022.1	Firmicutes	1	0	1986	11	4	10
**[Gram negative]**									
*Nostoc punctiforme*	PCC 73102	NC_010628.1	Cyanobacteria	0	1	6689	97	131	38
*Synechococcus sp*	CC9311	NC_008319.1	Cyanobacteria	0	1	2892	12	8	6
*Agrobacterium tumefaciens*	C58	NC_003062.2	Proteobacteria	0	1	5355	63	61	27
*Bartonella henselae*	Houston-1	NC_005956.1	Proteobacteria	1	0	1488	8	10	2
*Bradyrhizobium sp*	BTAi1	NC_009485.1	Proteobacteria	0	1	7621	98	103	51
*Escherichia coli* (Ec)	K-12 substr. MG1655	NC_000913.2	Proteobacteria	0	1	4146	32	24	18
*Helicobacter pylori*	83	CP002605.1	Proteobacteria	1	1	1609	5	1	7
*Klebsiella pneumoniae* (Kp)	342	NC_011283.1	Proteobacteria	1	0	5768	33	24	18
*Neisseria meningitidis*	MC58	NC_003112.1	Proteobacteria	1	0	2063	3	2	1
*Pseudomonas aeruginosa*	PAO1	NC_002516.1	Proteobacteria	1	1	5571	70	71	35
*Salmonella enterica*	*arizonae* serovar	NC_010067.1	Proteobacteria	1	1	4500	21	12	13
*Shigella flexneri*	2a str. 2457 T	NC_004741.1	Proteobacteria	1	0	4060	18	11	9
*Vibrio cholerae*	MJ-1236	NC_012668.1	Proteobacteria	1	1	3772	71	42	59
*Yersinia pestis*	CO92	NC_003143.1	Proteobacteria	1	1	4066	20	10	13
*Leptospira biflexa*	serovar Patoc	CP000788.1	Spirochaetes	0	1	3726	67	76	30
*Treponema pallidum*	*pallidum* SS14	CP000805.1	Spirochaetes	1	1	1028	5	1	6

^a^For those included in our phylogenetic analysis, abbreviations for species names are shown in parentheses.

^b^1: pathogenic, 0: non-pathogenic.

^c^1: motile, 0: non-motile.

^d^Total number of proteins in the genome.

^e^Total number of PAS/PDC-containing proteins identified in the genome.

^f^Total number of PAS domains identified.

^g^Total number of PDC domains identified.

^h^One of the PDC/PAS-containing proteins in *Staphylococcus haemolyticus* (Sh.3, YP_253148.1, PhoR) does not have the PDC domain identified by HHblits nor HHsearch, although all other *Staphylococcus* PhoR homologs have clearly identified PDC domains. However, as noted in Supplementary Table S1, a very weakly conserved PDC-like region was identified in this protein based on the predicted secondary structure. This potential PDC domain is not included in this table nor in our analysis.

Previously, a hypothesis was put forward that non-pathogenic bacteria have more PDC/PAS proteins than do pathogenic bacteria [1]. To test this hypothesis, we grouped bacteria based on its pathogenic status and determined if the two groups (pathogenic or not) have a significant difference in regard to the PDC/PAS protein numbers (see Table 1). The average numbers of PDC/PAS proteins were 18.9 for 30 pathogenic bacteria and 33.7 for 18 non-pathogenic bacteria. This difference was minimally significant (*p* = 0.048 by *t*-test, but *p* > 0.1 by non-parametric Mann–Whitney *U* test). When we removed the effect of over-representation of *Staphylococcus* genomes by removing 5 of the 8 genomes, all pathogenic (see Methods), no significant difference was observed (the average number of PDC/PAS proteins for 25 pathogenic genomes was 21.2; *p* > 0.1 by *t*-test and *p* > 0.3 by Mann–Whitney *U* test). Comparisons of the total protein numbers between the two pathogenicity groups had the same results. Although non-pathogenic bacteria tend to have more PAS domains, the non-significant difference suggests that acquisition and/or loss of PAS domains is largely independent of pathogenicity. In addition, these data suggest that another selectable phenotypic trait(s) is responsible for the variation in the number of PDC/PAS domains in bacteria.

One of the earliest bacterial PDC/PAS proteins identified in *Escherichia coli* was a methyl-accepting chemotaxis protein [27]. These proteins link changes in chemotactic effector concentrations with the flagella necessary to move bacteria toward or away from that effector. The involvement of PAS domains in chemotaxis led us to examine if a correlation existed between the number of PAS domains and bacterial motility. For this analysis, a bacterium was considered motile if any part of its life-cycle involved motility (Table 1). Average numbers of PDC/PAS proteins were highly significantly different between motile (43.8 for 19 genomes) and non-motile (12.7 for 24 genomes, excluding 5 *Staphylococcus* genomes) groups (*p* < 0.0001 by *t*-test). The mean number of the PDC/PAS proteins in the motile groups was approximately 4 times the mean in the non-motile group. Although variances were significantly different (*p* < 0.0001) between motility groups for the number of the PDC/PAS proteins, the test results were the same with and without the equal variance assumption. Using the non-parametric Mann–Whitney *U* test, where normal distribution of data can be violated, median numbers of PDC/PAS proteins as well as total proteins were highly significantly different between the motility groups (*p* < 0.0001). The average total numbers of proteins (3178.9 for non-motile and 4244.1 for motile groups) were also significantly, although weakly, different (*p* = 0.048 by *t*-test and *p* = 0.017 by Mann–Whitney *U* test). The test results were virtually the same when all 48 genomes were included in the analysis. While motility explains a substantial amount of the variation in the numbers of PDC/PAS as well as total proteins, the variation in the number of PDC/PAS proteins in bacteria must also involve other selectable phenotypes [28].

### PDC/PAS domain distribution in Gram-positive bacteria

**Figure 3:**
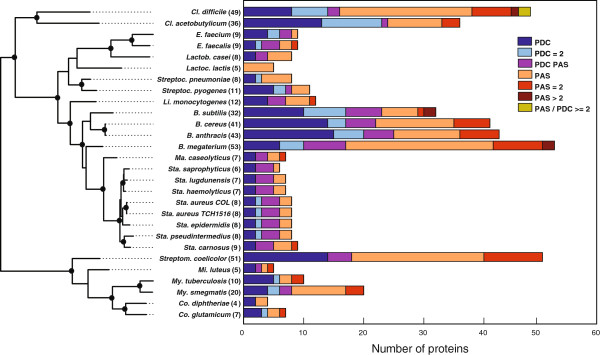
**Overview of PDC/PAS proteins in 28 Gram-positive bacteria.** The total number of PDC/PAS proteins found in each genome is shown in parentheses next to the species name and its corresponding bar length. Ranges of different colors in each bar indicate the proportions of different domain architectures. They include "single PDC" (dark blue: PDC), "two PDCs" (light blue: PDC = 2), "PDC and PAS" (purple: PDC PAS), "single PAS" (orange: PAS), "two PASs" (red: PAS = 2), "more than two PASs" (brown: PAS > 2), and "multiple PAS and PDC" (yellow green: PAS/PDC > =2). See (Additional file 1: Table S1) for the details. On the left hand side, the maximum likelihood phylogeny of 16S rDNA sequences is given to provide an evolutionary relationship among these bacteria. Dark circles at internal nodes represent those supported by bootstrap values greater than 70%.

To focus the scope of this study, we limited our detailed analysis of PDC/PAS proteins to Gram-positive bacteria because they represent major health challenges in the hospital and the community [29-31]. Similar to Gram-negative bacteria, the percentage of PDC/PAS proteins in Gram-positive bacteria as a function of the total number of proteins was low (0.97% in Gram-negative and 0.52% in Gram-positive bacteria). Also similar was that PDC/PAS proteins having a single PDC or PAS domain were the most common in both Gram-negative and Gram-positive bacteria (60.2% and 62.1%, respectively). One remarkable difference was that Gram-negative bacteria had approximately 4 times more PDC/PAS proteins that contained more than two PDC and/or PAS domains. Also consistent between Gram-negative and Gram-positive bacterial species was that the number of PDC/PAS proteins varied between genera and species (Table 1). There was also variation in the number of single and multiple PDC/PAS proteins between closely related bacteria (Figure 3, see also Additional file 1: Table S1). For example, *Streptococcus pneumoniae* and *Streptococcus pyogenes* have 8 and 11 proteins, respectively, that contain PDC and/or PAS domains. Two of the 11 PDC/PAS proteins in *S. pyogenes* contain two PDC domains (*i.e.*, 2 predicted YesM homologs: Spy.4/YP_598904.1 and Spy.5/YP_599031.1), while *S. pneumoniae* has one protein containing two PDC domains (*i.e.*, Spn.5/YP_816074.1, predicted to be a sensor histidine kinase). Similarly, *S. pyogenes* has one protein containing both one PDC and one PAS domains (*i.e.*, DpiB: Spy.3/YP_598556.1), while *S. pneumoniae* has none. Overall, our analysis found that Gram-positive bacteria tend to have fewer total PDC/PAS proteins than do Gram-negative bacteria and that Gram-positive PDC/PAS proteins infrequently have greater than 2 PDC/PAS domains. In addition, there was remarkable diversity within genera in the number of PDC/PAS proteins.

### Evolutionary relationships of PDC/PAS domains in Gram-positive bacteria

**Figure 4:**
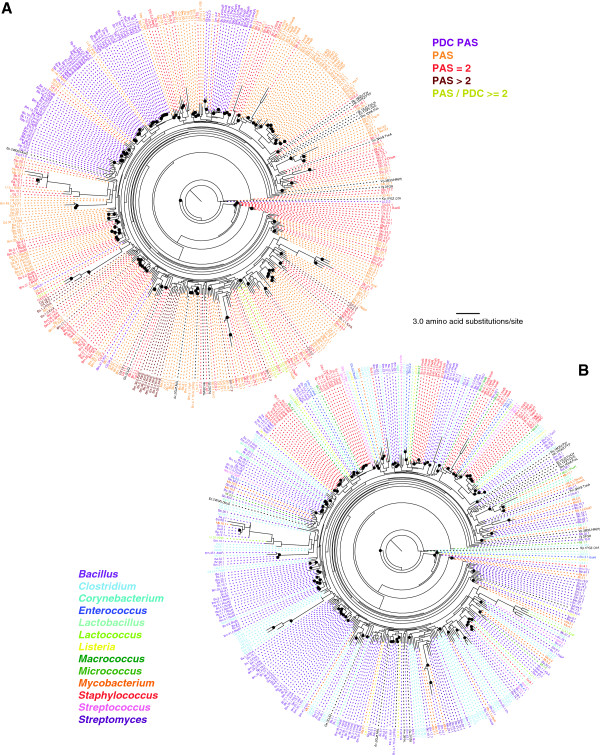
**Maximum likelihood phylogeny of PAS domain protein sequences.** 372 PAS domain sequences obtained from 28 Gram-positive bacterial genomes are included. As references, the phylogeny is shown in two color schemes: based on PDC/PAS domain architectures **(A)** and based on bacterial genera **(B)**. Black and green circles at internal nodes represent those supported by bootstrap values greater than 60% and those supported by all three phylogenetic methods although bootstrap values were 60% or lower. Fourteen PAS domain sequences whose structures are known and used as the search queries were included in the phylogeny and they are labeled with black letters. One PDC domain sequence, 1P0Z (CitA, *Klebsiella pneumoniae*), was also included as the outgroup and is shown in black. See Table 1 and (Additional file 1: Table S1) and (Additional file 3: Table S2) for the species name abbreviations and protein IDs. Species abbreviations not listed in Table 1 are Av (*Azotobacter vinelandii*), Bj (*Bradyrhizobium japonicum*), Gs (*Geobacter sulfurreducens*), Hm (*Haloarcula marismortui*), Hh (*Halorhodospira halophila*), Rc (*Rhodospirillum centenum*), Rj (*Rhodococcus jostii*), Rm (*Rhizobium meliloti*), Tm (*Thermotoga maritima*), Vp (*Vibrio parahaemolyticus*).

In order to understand the evolutionary relationships among PAS and PDC domain sequences in bacterial genomes, we performed phylogenetic analysis using each domain sequence separately. Figure 4 shows the maximum likelihood phylogeny based on PAS domains. As indicated with very short branch lengths, PAS domains are in general highly conserved. The phylogeny also shows that PAS domains from proteins with similar predicted functions, possibly orthologous proteins, tended to cluster together. As examples, the WalK/VicK/YycG orthologs (for simplicity, we will refer to these as WalK orthologs) are part of bacterial two-component systems that regulate cell wall biosynthesis and resistance to antibiotic stress [32-34]. The WalK ortholog proteins include either one PAS or one PAS and one PDC domain ("PDC PAS" proteins shown in purple in Figure 4A). The PDC domain found in WalK is the PAS-like fold identified by Szurmant *et al.* as being in the extracellular loop [35]. The PAS domains for these WalK orthologs form a large clade that encompasses those from all species examined. Similarly, the “PDC PAS” containing dicarboxylate sensors (*i.e*., DcuS, DctS, MalK and CitS) [18] cluster together, as do the sensor kinases ResE and SrrB of the two-component anaerobiosis regulatory systems ResDE and SrrAB [36-38]. Interestingly, in the PhoR cluster, we observed that the *Bacillus anthracis* PhoR protein contains only a single PAS domain. In other words, the PDC domain must have been lost during the evolution of the *Bacillus* lineage; however, their protein functions were likely retained. Another good example of PAS conservation can be found in proteins having two or more PAS domains. The two-component sensor histidine kinases, KinE, in *B. subtilis* (Bs.2) and *B. megaterium* (Bm.38) each have four PAS domains (Additional file 1: Table S1). All these PAS domains form a single cluster (shown in brown in Figure 4A), indicating that the duplication of these domains must have occurred prior to the divergence of these two species. Very different evolutionary relationships are found among multiple PAS domains identified in another kinase, KinA. The *B. subtilis* KinA has three PAS domains (shown in brown in Figure 4A) whereas the other *Bacillus* KinA proteins have only two PAS domains (shown in red). Only the first PAS domains of *B. authracis* (Ba.1.1), *B. cereus* (Bc.2.1), and *B. megaterium* (Bm.28.1), and the second PAS domains of *B. authracis* and *B. cereus* (Bc.2.2 and Ba.1.2) form clusters. While the second PAS domain of *B. megaterium* (Bm.28.2) and the second and third PAS domains of *B. subtilis* (Bs.1.2 and Bs.1.3) appear to share some similarity, the first PAS domain of *B. subtilis* (Bs.1.1) is highly diverged from any of these KinA PAS domains. This is interesting because this particular *B. subtilis* PAS domain (Bs.1.1) is involved in binding ATP and regulating the sporulation phosphorelay system [39]. Another interesting observation can be seen with two types of transcriptional regulators found in the *Bacillus* genomes, BkdR and RocR. BkdR proteins have two PAS domains (shown in red in Figure 4A) and RocR proteins have a single PAS domain (shown in orange in Figure 4A). The PAS domains of these proteins form a single cluster where the PAS domains of RocR are more similar to the second PAS domains of BkdR, illustrating a dynamic evolution of these PAS-containing transcriptional regulators. Specifically, after the duplication of PAS domains in the ancestral *Bacillus* BkdR proteins, the ancestral RocR protein was derived from the second PAS domain region of the BkdR protein. As both RocR and BkdR are involved in regulating amino acid catabolic pathways (arginine/ornithine and branched chain amino acids respectively) [40,41], this evolution may be linked to their functions.

**Figure 5:**
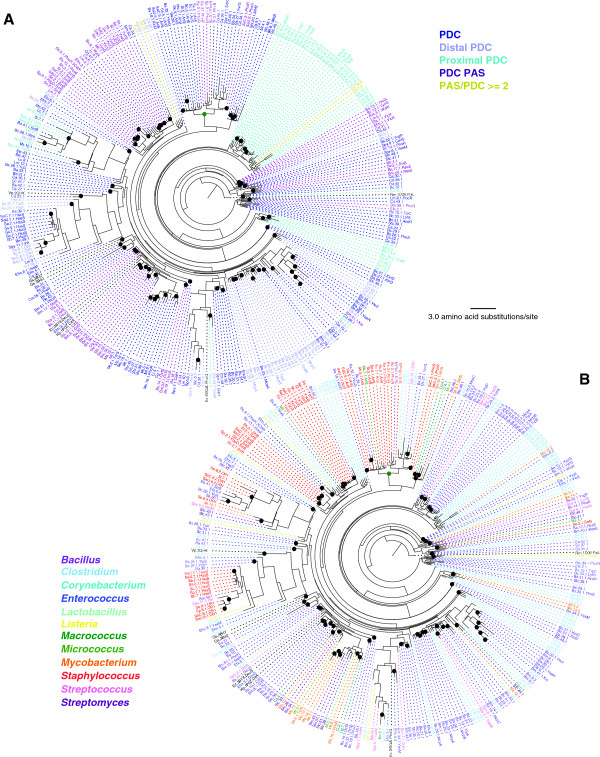
**Maximum likelihood phylogeny of PDC domain protein sequences.** 303 PDC domain sequences identified from 28 Gram-positive bacterial genomes are included. The phylogeny is shown in two color schemes: based on PDC/PAS domain architectures **(A)** and based on bacterial genera **(B)**. "Distal" and "Proximal" PDCs are the first and second, respectively, domains in the two-PDC proteins (shown as "PDC = 2" in Figure 3). Black and green circles at internal nodes represent those supported by bootstrap values greater than 60% and those supported by all three phylogenetic methods although bootstrap values were 60% or lower. Seven PDC domain sequences whose structures are known and used as the search queries were included in the phylogeny and they are labeled with black letters. One PAS domain sequence, 1D06 (FixL, *Rhizhobium meliloti*), was included as the outgroup and is shown in black. See Table 1 and (Additional file 1: Table S1) and (Additional file 3: Table S2) for the species name abbreviations and protein IDs. Species abbreviations not listed in Table 1 are Gs (*Geobacter sulfurreducens*)*,* Rm (*Rhizobium meliloti*)*,* and Vp (*Vibrio parahaemolyticus*).

Compared to the PAS phylogeny, PDC domains are significantly more divergent as indicated by the much longer branch lengths in Figure 5. Reflecting their higher degree of divergence, the clustering patterns shown in the PDC phylogeny are much more complex. For example, PhoR and WalK ortholog proteins, both are clustered individually in the PAS phylogeny (Figure 4), but are found in dispersed locations in the PDC phylogeny (*e.g.*, PDC domains from three *Bacillus* PhoR proteins: Bm.39.1 from *B. megaterium*, Bs.9.1 from *B. subtilis*, and Bc14.1 from *B. cereus*, are completely separated). Some proteins we examined contain two PDC domains and they are shown as "Proximal" and "Distal" in Figure 5A (in light blue and in light purple, respectively). Interestingly, the majority of the "Proximal" PDC domains form a single large cluster indicating their monophyletic origin. On the other hand, the "Distal" PDC domains are more divergent and show evolutionary patterns distinct from the "Proximal" domain.

In order to understand how phylogenies obtained from PAS and PDC domains individually correlate with the entire protein evolution, we used the WalK ortholog proteins as an example. We reconstructed three phylogenies from 17 WalK proteins: from PAS domain, from PDC domain, and from the entire protein sequences (see Additional file 2: Figure S1). For this analysis, the WalK orthologs from *Lactococcus lactis* and *Streptococcus* sp. were excluded due to the absence of a PDC domain in the extracellular loop. The entire WalK proteins are approximately 610 amino acid (aa) length, while their PAS and PDC domains are between 88–147 aa. The entire WalK protein phylogeny shows three clearly supported clusters: the WalK proteins from *Macrococcus caseolyticus* and the *Staphylococcus* sp., those from *Bacillus* sp. and *Listeria monocytogenes*, and those from *Lactobacillus casei* and *Enterococcus* sp. As described before, PAS domains of WalK proteins are much more conserved than their PDC domains, and their phylogenetic clustering was completely congruent with the entire protein relationship. In striking contrast, with the exception of the *Staphylococcus* domains, no clear clustering pattern was found in the PDC phylogeny. Taken together, these data suggest that PAS domain evolution is more conserved and protein/function-specific, while PDC domains are highly divergent and possibly contributed to a more dynamic protein function evolution.

### PDC/PAS domains of staphylococci

The medical importance of staphylococci and our interest in dicarboxylic acid sensing led us to examine the staphylococcal PDC/PAS proteins to gain insight into their potential function(s). As stated earlier, the number of PDC/PAS proteins correlates well with motility, such that motile bacteria have 4 times as many PDC/PAS proteins as do non-motile bacteria. The staphylococci are non-motile bacteria that are closely related to the motile bacterial species in the *Bacillus* genus (Figure 3). Based on the maximum likelihood phylogeny of the 16S rDNA sequences in Figure 3, when the staphylococcaceae (*i.e.*, *Macrococcus* and *Staphylococcus*) diverged from *Bacillus megaterium* it appears that motility was lost. Prior to that divergence, all of the related bacteria (*e.g.*, *Listeria monocytogenes* and *Bacillus* sp.) were/are motile (*B. anthracis* is a notable exception). Interestingly, the *Staphylococcus* sp. are most closely related to *Macrococcus caseolyticus*, which is a non-pathogenic and non-motile bacterium found in milk and on animal skin, suggesting that the transition from free-living bacterium to being host-adapted coincided with the loss of motility. The genus *Staphylococcus* is comprised of species that have between 6 (*i.e.*, *S. saprophyticus*) and 9 (*i.e.*, *S. carnosus*) proteins having one or more PDC/PAS domains, while the *Bacillus* genus contains species having between 32 (*i.e.*, *B. subtilis*) and 53 (*i.e.*, *B. megaterium*) PDC/PAS proteins (Figure 3 and Table 1). The loss of PDC/PAS proteins by staphylococci is consistent with the transition from a motile life-style to a non-motile life-style; however, the loss of motility is unlikely to be the complete explanation for the evolutionary loss of PDC/PAS proteins. A second phenotypic difference between the *Staphylococcus* and *Bacillus* genera is the ability to sporulate, with the *Bacillus* sp. able to form spores but not the staphylococci. Four of the kinases (*i.e*., KinA, KinC, KinD, and KinE) that regulate sporulation in *Bacillus* sp. [42] all contain PDC/PAS domains (see Additional file 1: Table S1). In contrast to the *Bacillus* sp., staphylococci have an oxygen-sensing two-component system (NreB-NreC) that regulates transcription of the nitrate and nitrite reductase systems [43,44]. NreB, the sensor histidine kinase portion of the two component system, uses a PAS domain to coordinate binding of an oxygen-labile [4Fe-4S]^2+^ iron-sulfur cluster. Lastly, it was shown that the number of PDC/PAS proteins correlates with the presence of electron transport proteins [28]. *Bacillus* sp. and *Staphylococcus* sp. all possess active electron transport systems; hence, the difference in the number of PDC/PAS proteins is unlikely to be due to the changes in electron transport. In total, these observations suggest the transition from a free-living bacterium to a host-adapted bacterium coincided with the loss of most PDC/PAS proteins from the *Staphylococcus* genus through reductive evolution.

While reductive evolution likely led to the loss of many PDC/PAS proteins in staphylococci, all staphylococci in this study maintained orthologs of six PDC/PAS proteins; specifically, WalK (shown as *yycG* or VicK in Additional file 1: Table S1), SrrB, PhoR, ArlS, HssS, and GdpP. Five of these proteins are members of two component signal transduction systems that are important for oxygen-sensing (SrrB) [38], response to phosphate-limited conditions (PhoR) [45], regulation of hemin transport (HssS) [46], autolysis (ArlS) [47], and the regulation of cell wall biosynthesis (WalK) [48,49]. The sixth conserved PDC/PAS protein, GdpP, has recently been described as being a cyclic-di-AMP phosphodiesterase that suppresses a mutation in *S. aureus* lipoteichoic acid biosynthesis [50]. Importantly, these sensory proteins not only regulate physiologically essential processes, but they also influence staphylococcal virulence and antibiotic resistance [34,38,46,51,52]. Although these six proteins are conserved in staphylococci, the evolutionary divergence of the PDC domains can be seen in staphylococci where a weakly conserved PDC-like region was identified in the ortholog of PhoR from *S. haemolyticus* and the HssS of *S. lugdunensis* (YP_003470952.1) lacks the PDC domain.

As mentioned before, all staphylococci included in this study, with the exception of *S. saprophyticus*, have NreB orthologs (see Additional file 1: Table S1). Similarly, four additional PDC/PAS proteins were identified that are present in some *Staphylococcus* species and whose functions are unknown. *S. aureus*, *S. epidermidis* and *S. pseudintermedius* have a protein containing two PDC domains (*i*.*e*., SaC.8/YP_185101.1, SaU.8/YP_001574141.1, Se.8/NP_763721.1, Sp.8/YP_004150417.1; listed as OR1 in Additional file 1: Table S1), *S. lugdunensis* has a PDC domain containing protein (*i*.*e*., Sl.7/ ADC86684.1), and *S. carnosus* has two PAS domain containing proteins (*i.e.*, Sc.8/YP_002635456.1 and Sc.9/YP_002634788.1). The two PDC domains of the OR1 orthologs in *S. aureus*, *S. epidermidis* and *S. pseudintermedius* cluster in two separate clades: the “Distal” PDC domains are close to the PDC domains of HssS that is involved in regulating hemin transport, while the “Proximal” PDC domain forms a cluster that also contains the single PDC domain of Sl.7 (see Figure 5). To gain more insight into the potential functions of these proteins, the protein sequences were analyzed by Gene Ontology (GO) and BLASTP/PSI-BLAST similarity search. These analyses revealed both proteins as having similarities to histidine kinases with “two–component sensor activity” (GO:0000155) and containing typical domains of histidine kinases (*i.e.* HisKa and HATPase). The possibility that both proteins are sensory histidine kinases of two component systems is supported by the presence of putative cognate response regulators adjacent to the corresponding genes. Interestingly, the genes coding for the OR1 proteins and their cognate response regulators are predicted to be co-transcribed with an upstream gene that is predicted to code for ABC transporter iron-binding proteins. Taken together, the genomic organization and the close phylogenetic relation of the OR1 PDC domain with that of HssS, suggest that OR1 may function in regulating iron homeostasis.

GO analysis of the *S. carnosus* Sc.8/YP_002635456.1 (found in Additional file 1: Table S1) protein, which contains two PAS domains, suggests it has two-component system sensor kinase activity, with some of the top BLASTP/PSI-BLAST hits from *Bacillus* sp. being an ethanolamine two-component sensor histidine kinase. Consistent with this suggestion, there is a putative ethanolamine transporter (ORF Sca_2366) located adjacent to the genes coding for the putative two component system. The PAS domain of the second PDC/PAS protein of unknown function in *S. carnosus* (*i.e.*, Sc.9/YP_002634788.1) belongs to a highly supported cluster including six other single-PAS containing proteins (Ll.1/YP_001032211.1, Ca.1/NP_348158.1, Lc.3/YP_805881.1, Spn.4/YP_816768.1, Efs.4/NP_814954.1, and Spn.1/YP_815994.1; Figure 4). Two of the PAS domains in this cluster, Lc.3/YP_805881.1 and Spn.4/YP_816768.1, are annotated as flavoproteins having oxidoreductase activity. The top hits in PSI-BLAST for Sc.9/YP_002634788.1 all have a sensory box in the C-terminus that is predicted to be a hemerythrin-binding domain. Hemerythrin-binding proteins are non-heme oxygen-binding proteins that are found in marine invertebrates (*e.g.*, brachiopods) and some bacteria. Interestingly, in Firmicutes, hemerythrin-binding proteins are primarily present in anaerobic bacteria, such as the Clostridia; thus, it may be that Sc.9/YP_002634788.1 is important for *S. carnosus* in the adaptation to an anaerobic environment.

## Conclusions

Bacterial evolution has led to huge variations in the number of PDC/PAS proteins within all genera. Several hypotheses have been put forth to explain this variation as associated with pathogen status, genome size, or the presence of electron transport proteins. Undoubtedly, all of these bacterial traits contribute to variation in the number of PDC/PAS proteins; however, our analysis suggests that the number of PDC/PAS domains correlates to bacterial motility. This observation partially explains the selective loss of PDC/PAS domains in the genus *Staphylococcus* after its divergence from the genus *Bacillus*.

## Methods

### Bacterial genomes

48 bacterial genomes from the National Center for Biotechnology and Information (NCBI) [53] were chosen so as to include pathogenic and non-pathogenic bacteria, commensal bacteria, and saprophytes (Table 1). Of the 48 genomes, 32 are from Gram-positive bacteria belonging to the Firmicute and Actinobacteria phyla and representing 14 genera (*Clostridium*, *Streptococcus*, *Enterococcus*, *Listeria*, *Lactobacillus*, *Lactococcus*, *Bacillus*, *Staphylococcus*, *Mycobacterium*, *Corynebacterium*, *Micrococcus*, *Streptomyces*, *Macrococcus*, and *Bifidobacterium*). For the detailed study of staphylococci, the genomes of seven different species and two strains of *Staphylococcus aureus subsp. aureus* (*i.e.*, COL and USA300 TCH1516) were selected. In addition, sixteen Gram-negative bacterial genomes representing Alpha, Gamma, and Epsilon proteobacteria, two Spirochaetes (*Leptospira* and *Treponema*), and two Cyanobacteria (*Synechococcus* and *Nostoc*) were included in the analysis.

### PDC/PAS domain search methods

Due to the extreme divergence (< 20% identity) of PDC/PAS domain sequences, sequence similarity search methods such as Basic Local Alignment Search Tool (BLAST) [23,54] or FASTA [55] are minimally effective at identifying PDC/PAS proteins. Profile or profile HMM methods such as PSI-BLAST [23] and HMMER [56] have been more successful at identifying PDC/PAS proteins (*e.g.*, [24]). More recently, HMM-to-HMM comparisons were used by Chang *et al.*[19] to identify PDC domains in *Bacillus subtilis*. In order to increase the likelihood of identifying PDC/PAS proteins as thoroughly as possible, we chose to use HHblits (HH-suite 2.0.14) [26], the current state-of-the-art method. Although HHblits is in general highly sensitive, due to its use of prefilters and discretization of database HMMs [26], occasionally some sequences can be missed by HHblits. In order to complement the results of HHblits, we also used HHsearch (ver. 1.5.1) [57]. Both packages were downloaded [58] and installed on a local Linux server. The methods require profile HMMs constructed from queries (PDC/PAS domain sequences) as well as from protein sequences included in the bacterial genome databases. Secondary structure information of each sequence was also added to the HMMs. Additional details of the search methods are described in the following sections.

### PDC/PAS domain queries

3D-structural information from 86 PDC/PAS domain sequences (60 PAS and 26 PDC domains; see in Additional file 3: Table S2) were downloaded from the Protein Data Bank (PDB) [59,60]. These structures represented eight bacterial phyla that contained proteins with diverse ligand affinities. When proteins have multiple domains, PAS or PDC domains were extracted and used as queries individually.

### Preparation of HMM queries and HMM databases

In order to perform HMM-to-HMM comparisons, both query sequences and protein sequences in each bacterial genome needed to be converted to HMMs. For HHblits, this was done by using the programs hhblits, addss.pl, and hhmake, all using the default settings. The program hhblits was used to iteratively search the non-redundant database (nr20, included in the HH-suite 2.0.14) with a protein sequence from the queries or from the bacterial genomes. The options were set as default, except to perform three iterations with the E-value threshold for inclusion at 0.001. From each iteration, sequences within the inclusion threshold were added to the query or to its multiple sequence alignment (MSA). The script addss.pl was then used to incorporate the secondary structure information predicted by PSIPRED (ver. 3.2) [61] to the MSA generated for each protein sequence. Finally, the program hhmake was used to generate the HMM from each MSA. To generate the final HHblits databases for the bacterial genome data sets, the program hhblitsdb.pl was also used. For HHsearch, the overall process taken was similar to what described for HHblits. In order to build MSAs for query sequences, the program builali.pl was used, which runs PSI-BLAST (ver. 2.2.17; inclusion E-value threshold = 0.001 and 8 iterations) against the filtered NCBI non-redundant protein database obtained from HHsearch 1.5.1.

### PDC/PAS domain sequence search against bacterial genomes

The PDC/PAS domain sequence search was done with hhblits and hhsearch programs using each domain HMM query against each bacterial HMM database prepared above. The same settings were used for iteration numbers and inclusion E-value thresholds. For both HHblits and HHsearch, the hits were considered as significant based on the E-value threshold of 1, 95% probability (this is "the probability of template to be a true positive" calculated by HHblits), and the length of aligned HMM regions to be 90 amino acids. Since known PDC/PAS domains are 100–140 amino acid long (see Additional file 3: Table S2), 90 amino acids cover 65-90% of the domain. This alignment coverage gave us sufficiently strong search results. When HHblits did not give a significant hit but HHsearch did, the hit by HHsearch was also included. In some cases, we also found significant hits shorter than 90 amino acids whose E-values and probabilities were within our threshold. We examined these cases for a) their alignment quality, b) if PAS/PDC topology is recognized from the entire protein, and c) if their orthologs in other species have a PAS/PDC domain. We identified 11 cases to be positive based on these further analyses. These HHsearch-based as well as short PAS/PDC domains are marked in (Additional file 1: Table S1). All bacterial protein HMM databases and query HMMs used for this study are available from: http://bioinfolab.unl.edu/emlab/PAS_PDC.

### Classification of PDC/PAS domains

All the significant PDC/PAS domains identified from HHblits and HHsearch were classified based on their significant similarities with either PAS or PDC query. As described before, PAS and PDC domains are defined, respectively, with α-2β-4α-3β and 3α-2β-1/2α-3β-α topologies (see the examples in Additional file 4: Figures S2 and S3). All our identified hits within the threshold followed these secondary structure patterns. Note that GAF domains (named for their presence in cyclic GMP-specific phosphodiesterase, adenylyl cyclase, and FhlA), for example, share a similar folding pattern (α-3β-α-β-α-2β-α); however, using a conservative threshold with HHblits/HHsearch, our results do not include GAF-domain containing proteins. All identified PAS and PDC domains are listed in (Additional file 1: Table S1). For convenience, we labeled all PDC/PAS proteins using abbreviations of species name and numbers (*e.g.*, Cd.1 for the first protein of *Clostridium difficile*). All PDC and PAS domains of each protein are then numbered in the order of their occurrence (*e.g.,* Cd.1.1 and Cd.1.2 for the two PAS domains identified in the protein Cd.1). These domain names are used in all alignments and phylogenies.

### Multiple sequence alignment

All identified PAS and PDC domains were individually aligned using PRALINE [62]. BLOSUM45 amino acid substitution matrix, open and extension gap penalties of 12 and 1, respectively, PSI-BLAST against the protein non-redundant database with 3 iterations and E-value threshold = 0.01, DSSP structural features, and PSIPRED secondary structure prediction were chosen from the options. The MSAs of all identified PAS and PDC domains with their predicted secondary structures are available from: http://bioinfolab.unl.edu/emlab/PAS_PDC.

For bacterial 16S ribosomal DNA (rDNA) sequences, the alignment was generated using MAFFT v6.847b [63] with the L-INS-i algorithm. This MSA is also available from our website.

### Phylogenetic tree reconstruction

The maximum-likelihood phylogenies for the PAS and PDC domains were reconstructed using RAxML version 7.0.4 [64]. All the options were set to default except for PROTGAMMAJTT for the –m option, which estimates the gamma model for rate heterogeneity and uses the JTT amino acid substitution model. We also used two other phylogenetic methods, FastTree (ver. 2.1.4) [65] and Neighbor-Joining (PHYLIP 3.69) [66]. For both methods, the options for the gamma model of rate heterogeneity and the JTT amino acid substitution model were used. Bootstrap analysis was done with 1000 pseudoreplicates for all phylogenetic analyses. The program consense from the PHYLIP package was used to estimate the consensus phylogeny as well as the bootstrap values. In addition to bootstrap supporting values, we also calculated the supporting values for internal branches by three phylogenetic methods (3 as the highest support). For the 16S rDNA phylogeny, the maximum-likelihood phylogeny was reconstructed using PhyML version 3.0 [67]. All options were set to default except for estimating the gamma distribution parameter. Bootstrap analysis was done with 1000 pseudoreplicates.

### Ortholog and gene ontology analysis of *Staphylococcus* proteins

Orthology of all PDC/PAS proteins identified from *Staphylococcus* genomes was determined using reciprocal best-hit analysis in a BLAST similarity search. In this analysis, an unknown PDC/PAS protein from a *Staphylococcus* species was first used as a query for a protein similarity search using the blastp program against the *S. aureus* COL genome. The top hit protein in *S. aureus* was then used as the blastp search query against the original *Staphylococcus* genome. If the top hit from this second search was the original query protein, the query protein in the original species and the *S. aureus* protein are bi-directional or reciprocal best hits and considered to be coded by orthologous genes. When the *S. aureus* ortholog was not identified as a known gene, reciprocal blast analysis was performed also using other *Staphylococcus* genomes. Besides orthology analysis, functions of *Staphylococcus* PDC/PAS proteins were also inferred by BLAST similarity search against the Gene Ontology database using AmiGo [68,69].

### Statistical analyses

All statistical analyses were done using JMP v10.0.2 (SAS Institute Inc.). Our dataset included eight genomes of closely related *Staphylococcus* species; hence, there was a possibility that over-representation of staphylococcal genomes could skew our data analysis. To address this possibility, all statistical analyses were done twice; once using the entire data set from all 48 bacterial genomes, and once using the data from only 43 bacterial genomes, which had only three representatives from the eight *Staphylococcus* genomes: *S. aureus* TCH1516, *S. saprophyticus*, and *S. carnosus*.

## Competing interests

The authors declare that they have no competing interests.

## Authors’ contributions

RG and GAS conceived of the project. NS, RG, ENM, HM, and GAS contributed to the experimental design and execution. ENM and KME performed the statistical analyses. NS, RG, ENM, HM, KME, and GAS wrote the manuscript. All authors read and approved the final manuscript.

## Supplementary Material

Additional file 1: Table S1 All PDC/PAS-containing proteins identified in this study. Click here for file

Additional file 3: Table S2 PDB entries used for PAS or PDC queries. Click here for file

Additional file 2: Figure S1 Maximum likelihood phylogenies of WalK genes. Click here for file

Additional file 4: Figure S2 An example multiple sequence alignment of PAS domains. **Figure S3**. An example multiple sequence alignment of PDC domains. Click here for file
