# Supplementary material for: Reductive evolution and the loss of PDC/PAS domains from the genus Staphylococcus
Source: BMC Genomics. 2013 Jul 31;14:524. doi: 10.1186/1471-2164-14-524 (PMC3734008; doi:10.1186/1471-2164-14-524)
Supplement: Additional file 2: Figure S1 — Maximum likelihood phylogenies of WalK genes. [file 1471-2164-14-524-S2.pdf]

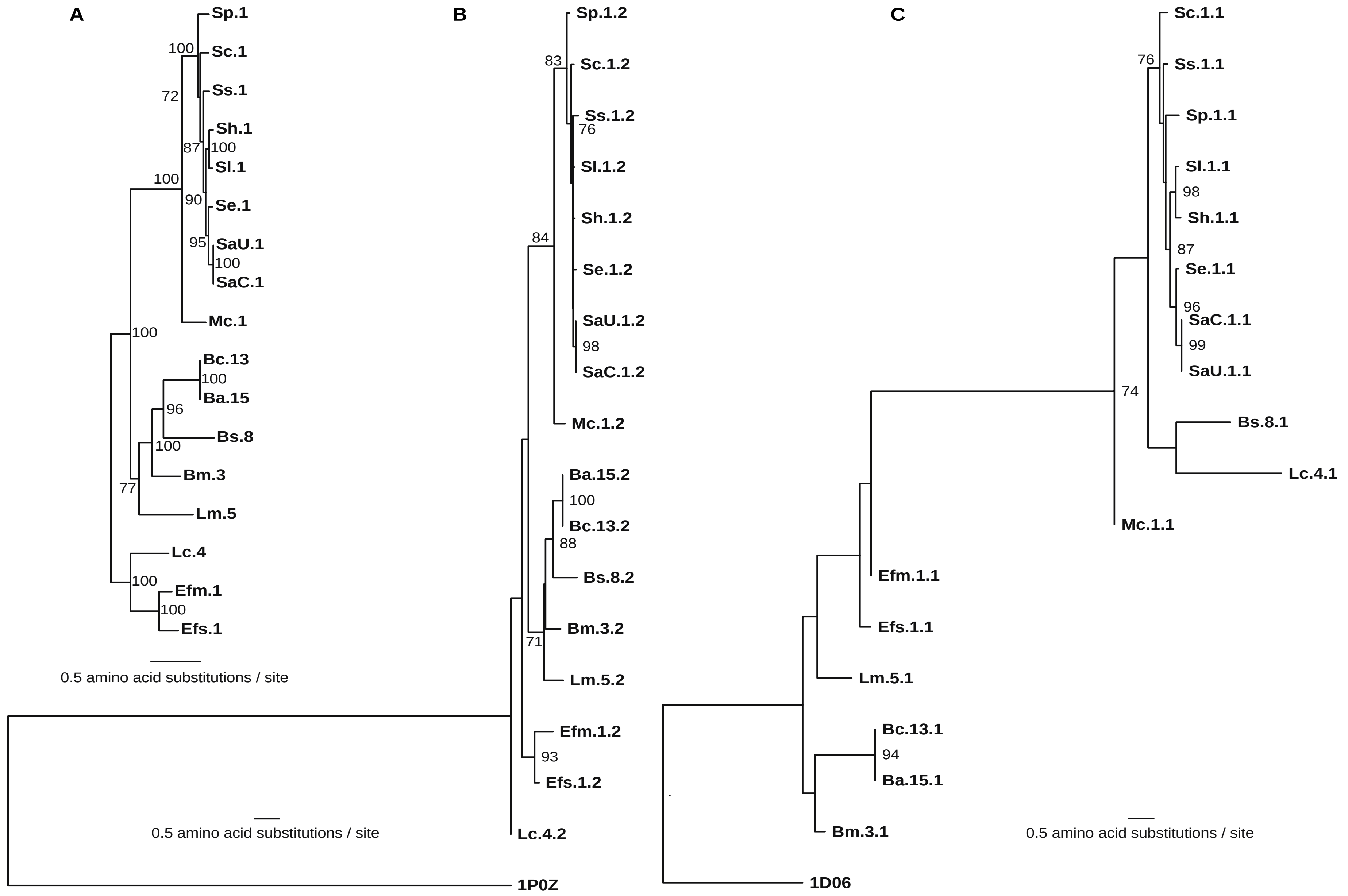

Figure S1: Maximum likelihood phylogenies of WalK proteins. For 17 WalK ortholog proteins, alignments of the entire protein sequences (A), the PAS domain sequences (B), and the PDC domain sequences (C) are used to reconstruct the phylogenies. Bootstrap support values greater than 70% are shown at internal nodes. Refer to Table 1 and Table S1 (Additional file 1) for the species abbreviations and protein/domain labels.
