# Supplementary material for: Reductive evolution and the loss of PDC/PAS domains from the genus Staphylococcus
Source: BMC Genomics. 2013 Jul 31;14:524. doi: 10.1186/1471-2164-14-524 (PMC3734008; doi:10.1186/1471-2164-14-524)
Supplement: Additional file 4: Figure S2 — An example multiple sequence alignment of PAS domains. Figure S3. An example multiple sequence alignment of PDC domains. [file 1471-2164-14-524-S4.pdf]

```
                α1                        β1              β2        α2            α3
2W0N       GHMGLEPYEISTLFEQR---QAMLQSIKEGVVAVDD-RGEVTLINDAAQELLNY---RKS
2GJ3       -------------ELLPEIFRQTVEHAPIAISITDL-KANILYANRAFRTITG-Y-GSEE
3B33       -SNAMDTSLPSAILNNMV----------TATLILDDG-LAIRYANPAAELLFS-QS-AKR

              α4                  α5                β3                    β4
2W0N       QDDEKLSTLSHS--WSQVV-DVSEVLRD----GTPRRDEEITIK---D---RLLLINTVP
2GJ3       VLGKNESIL-SN--GTTPRLVYQALWGRLAQKKPWSGVLVNRR--KDK-TLYLAELTVAP
3B33       IVEQSLSQLI--QHASLDLALLTQPLQSGQSI----TDSDVTFVVD--GRPLMLEVTVSP

                  β5
2W0N       VRSN-GVIIGAISTFRDKT---
2GJ3       VLNEAGETIYYLGMHRDTSELH
3B33       ITWQRQ--LMLLVEMRKID---
```

Figure S2: An example multiple sequence alignment of PAS domains. Sequences are labeled with their PDB identifiers. The alignment was generated using PRALINE. α-helix and β-strand assignments obtained from each PDB entry are marked in red and blue shades, respectively, and they are labeled above the alignment. Sequences used are: 2W0N (DcuS, *Escherichia coli*), 2GJ3 (NifL, *Azotobacter vinelandii*), and 3B33 (NR(II), *Vibrio parahaemolyticus*). The entire alignment including all identified PAS domain sequences with their secondary structures is available from: http://bioinfolab.unl.edu/emlab/PAS_PDC

```
                       α1                  α2                  α3
3BY8       GSSQISDMTRDGLANKALAVARTLADSPEIRQGLQKKPQESGIQAIAEAVRKRNDLLFIV
1P0Z       -----EERLHYQVGQRALIQAMQISAMPELVEAVQKRD-LARIKALIDPMRSFSDATYIT
3B42       ------RSSLDLQLKNARNLAGLII--HDIDGYMMKGD-SSEVDRFISAVKSKNFIMDLR

            β1      β2       α4            α5            β3               β4
3BY8       VTDMQSLRYSHPEAQRIGQPFKGDDILKALN-GEENVAINRGFLAQALRVFTPIYDENH-
1P0Z       VGDASGQRLYHVNPDEIGKSMEGGDSDEALINAKSYVSVRKGSLGSSLRGKSPIQDATG-
3B42       VFDEQAKEVSPTPSQTPNAKI----QQAIAAGRTLEFKET-LDGKRTLSLVLPFPNEQRC

                            β5           α6
3BY8       --------KQIGVVAIGLELSRVTQQINDSRW
1P0Z       --------KVIGIVSVGYTIEQLEHH------
3B42       QSCHDAGAAYLGGLLVTTSIEEGYEGARH---
```

Figure S3: An example multiple sequence alignment of PDC domains. Sequences are labeled with their PDB identifiers. The alignment was generated using PRALINE. α-helix and β-strand assignments obtained from each PDB entry are marked in red and blue shades, respectively, and they are labeled above the alignment. Sequences used are: 3BY8 (DcuS, *Escherichia coli*), 1P0Z (CitA, *Klebsiella pneumoniae*), and 3B42 (Mcp40H-20, *Geobacter sulfurreducens*). The entire alignment including all identified PDC domain sequences with their secondary structures is available from: http://bioinfolab.unl.edu/emlab/PAS_PDC
